# Polysaccharide CM1 from *Cordyceps militaris* hinders adipocyte differentiation and alleviates hyperlipidemia in LDLR^*(+/−)*^ hamsters

**DOI:** 10.1186/s12944-021-01606-6

**Published:** 2021-12-13

**Authors:** Wen-Qian Yu, Fan Yin, Nuo Shen, Ping Lin, Bin Xia, Yan-Jie Li, Shou-Dong Guo

**Affiliations:** Institute of Lipid Metabolism and Atherosclerosis, Innovative Drug Research Centre, School of Pharmacy, Weifang Medical University, Baotongxi street 7166#, Weifang, Shandong province China

**Keywords:** Polysaccharide, Hyperlipidemia, Lipid homeostasis, NPC1L1, Adipocyte

## Abstract

**Background:**

*Cordyceps militaris* is cultured widely as an edible mushroom and accumulating evidence in mice have demonstrated that the polysaccharides of *Cordyceps* species have lipid-lowering effects. However, lipid metabolism in mice is significantly different from that in humans, making a full understanding of the mechanisms at play critical.

**Methods:**

After 5 months, the hamsters were weighed and sampled under anesthesia after overnight fasting. The lipid-lowering effect and mechanisms of the polysaccharide CM1 was investigated by cellular and molecular technologies. Furthermore, the effect of the polysaccharide CM1 (100 μg/mL) on inhibiting adipocyte differentiation was investigated in vitro.

**Results:**

CM1, a polysaccharide from *C. militaris*, significantly decreased plasma total cholesterol, triglyceride and epididymal fat index in LDLR^*(+/−)*^ hamsters, which have a human-like lipid profile. After 5 months’ administration, CM1 decreased the plasma level of apolipoprotein B48, modulated the expression of key genes and proteins in liver, small intestine, and epididymal fat. CM1 also inhibited preadipocyte differentiation in 3T3-L1 cells by downregulating the key genes involved in lipid droplet formation.

**Conclusions:**

The polysaccharide CM1 lowers lipid and adipocyte differentiation by several pathways, and it has potential applications for hyperlipidemia prevention.

**Supplementary Information:**

The online version contains supplementary material available at 10.1186/s12944-021-01606-6.

## Background

Hyperlipidemia is a pathogenic factor for cardiovascular disease (CVD) [1–3]. Presently, cholesterol-lowering drugs, such as statins and proprotein convertase subtilisin/kexin type 9 (PCSK9) antibodies, play important roles in prevention and treatment of CVD [1–3]. Furthermore, ezetimibe has been developed to inhibit cholesterol reabsorption in the gut lumen by targeting Niemann-Pick C1-like 1 (NPC1L1) [4–6]. However, the currently deployed lipid-lowering drugs are unable to completely retard the progression of CVD. In more recent years, significant research has been conducted aimed at utilizing natural compounds in food for the same purposes, due to their potentially lower toxicity levels.

The edible fungus *Cordyceps militaris* is a commercialized mushroom that primarily consumed in Asian countries [7–9], and it is often used as a soup ingredient in South China. This region of China also has a lower mortality of atherosclerotic CVD per 100,000 compared with that of North China (65.0 vs 121.2) [1]. Although there is no direct evidence that the fruiting body of *C. militaris* can decrease atherosclerotic CVD in humans, a significant amount of evidence in mice has demonstrated that the water extracts of *Cordyceps* species have various bioactivities, including anti-hyperlipidemia and anti-atherosclerosis effects [8–10]. Based on the available data, the crude extracts rather than purified polysaccharides of *Cordyceps* species are generally used in the previous in vivo studies [11–14]. Furthermore, lipid metabolism in mice is different from that of humans, primarily due to the lack of cholesteryl ester transfer protein (CETP) in mice and distinct apolipoprotein (apo) B editing [15]. Additionally, the treatment time in the previous studies is generally less than 2 months. As hyperlipidemia needs a long-term intervention, it is necessary to clarify the lipid-lowering effects and mechanisms of these polysaccharides in animals whose lipid profiles are closer to humans based on a longer intervention time.

**Fig. 1:**
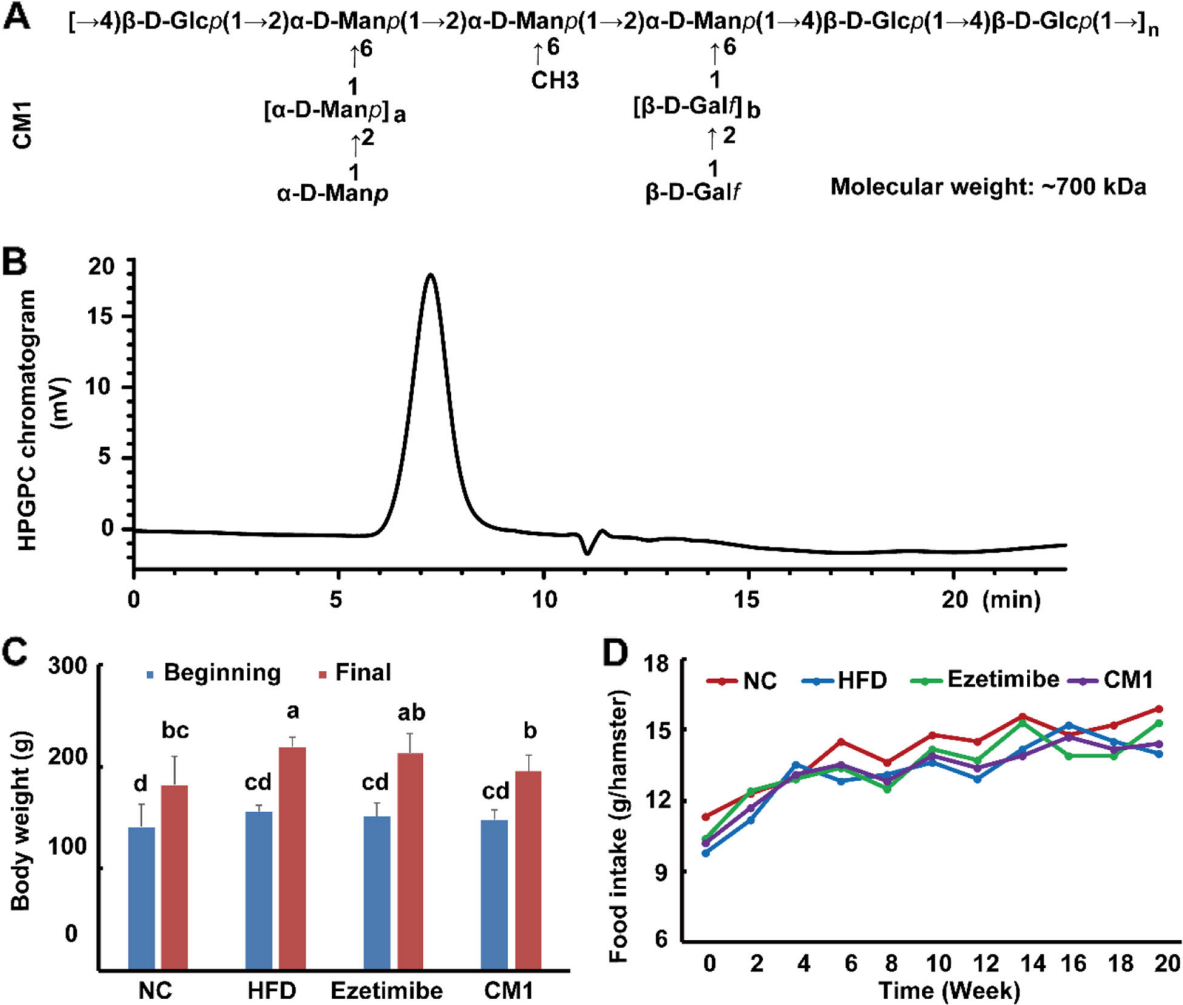
Structure and purity of CM1, and body weight and food intake of the LDLR(+/-) hamsters. A, presumed structure of polysaccharide CM1, *n* ≈ 390; B, purity of the polysaccharide CM1; C, body weigh changes of the LDLR^*(+/−)*^ hamsters in each group (*n* = 5); D, the average food intake of the LDLR^*(+/−)*^ hamsters in each group. NC: the regular chow diet group; HFD: the high-fat diet group. Different letters represent significant differences at a *P* < 0.05, and the same letters represent there is no significant difference between two groups. All the abbreviations and statistical letters are suitable for the rest figures

Hamsters have CETP and a low level of hepatic cholesterol synthesis similar to those seen in humans, making them excellent models for studies involving lipid metabolism [15]. Recently, researchers successfully established low-density lipoprotein receptor (LDLR)-deficient (LDLR)^*(−/−)*^ and heterozygous LDLR-deficient (LDLR^*(+/−)*^) hamster models, which have an autosomal inherited hypercholesterolemia [4, 15]. Hepatic LDLR is mainly responsible for the clearance of apoB-containing particles in circulation [16], making LDLR^*(+/−)*^ hamsters ideal for studying bioactive compounds with anti-hyperlipidemic activities. In a previous study, the polysaccharide CM1, mainly consisted of →4)-β-D-Glc*p* (1 → and →2)-α-D-Man*p* (1 → glycosyls (Fig. 1), with cholesterol efflux promoting activity was purified from the fruiting body of *C. militaris* [17]. In this study, LDLR^*(+/−)*^ hamsters, whose lipid profiles are similar to those seen in humans, were used to explore the effect of long-term treatment of CM1 in attenuating hyperlipidemia and regulating lipid metabolism-related genes and proteins.

## Materials and methods

### Materials

CM1 was obtained from *C. militaris* as the previously described method (Fig. 1A and B) [17]. Ezetimibe was the product of Selleck (Shanghai, China). The murine 3T3-L1 cell line was bought from National Collection of Authenticated Cell Cultures (Shanghai, China). Dexamethasone, 3-isobutyl-1-methylanxthine, and insulin were the products of Sigma-Aldrich (St. Louis, MO, USA). Dulbecco’s modified Eagle’s medium (DMEM) and fetal bovine serum (FBS) were the products of Thermo Fisher Biochemical Products (Beijing) Co. Ltd. (Beijing, China). Assay kits for lipid analysis and lipoprotein lipase (LPL) activity were obtained from Biosino Bio-technology and Science Inc. (Beijing, China). Lipofectamine RNAiMAX was bought from Invitrogen (Lot: 2164333, CA, USA). Opti-MEM was obtained from Gibico (Grand Island, NY, USA). Hematoxylin and eosin working solutions were bought from Nanchang YULU Laboratory Equipment Co., Ltd. (Jiangxi, China).

### Cell culture and treatment

Cells were cultured as the previously described method [18]. At day one, cells were set in six-well dishes at a density of 2 × 10^5^ cells per well. At day two, cells were separated into different groups: blank, differentiated, and CM1 group (100 μg/mL). The in vitro dosage of CM1 was determined according to the previous studies [17, 19]. The cells in the blank group were treated with the basal medium, and the medium was changed every 48 h. The cells in the differentiated group and CM1 group were treated with differentiation medium I (basal medium containing 4.0 μg/mL insulin, 0.5 mM 3-isobutyl-1-methylanxthine, and 1.0 μM dexamethasone) and differentiation medium I plus CM1, respectively, for 2 days. At day four, the cells, except for the blank group, were treated with differentiation medium II (basal medium containing 4.0 μg/mL insulin) and differentiation medium II plus CM1, respectively, for 2 days. At day six, the medium in each group was changed to the basal medium. At day eight, cells were labeled with BODIPY (493/505) or collected for quantitative RT-PCR analysis. For in situ staining, cells were labeled with 1.0 μM BODIPY [20].

Furthermore, 3T3-L1 cells that seeded in six-well dishes were transfected with peroxisome proliferator-activated receptor (PPAR) α siRNA (target sequence: 5′-GGAGCATTGAACATCGAAT-3′). The target sequence for scrambled siRNA was not provided by the company due to patent protection. Cells were transfected with scrambled or PPARα siRNAs (10 nM) using Lipofectamine RNAiMax (3 μL per well) that dissolved in Opti-MEM according to the instruction. Cells were then treated with or without CM1 (100 μg/mL) for another 24 h.

### Animal grouping and treatment

This study was approved by Weifang Medical University (2020SDL106–3). Twenty LDLR^*(+/−)*^ hamsters (male, 152 ± 14 g) were provided by Peking University (Beijing, China). As a bioactive macromolecule, CM1 may affect the gut system rather than directly act on the circulation. Therefore, ezetimibe, a lipid-lowering drug which acts on the internalization of NPC1L1 in intestine [5, 6], was used as a positive control. Except for the regular chow (NC) group, LDLR^*(+/−)*^ hamsters in the high-fat diet group (HFD), the ezetimibe group (25 mg/kg/d), and the CM1 group (CM1, 100 mg/kg/d) were fed a high-fat diet (0.15% cholesterol). The dosages of ezetimibe and CM1 were determined according to the available purified polysaccharide and the previous studies [4, 10, 21]. After 5 months, the hamsters were weighed and sampled under anesthesia after overnight fasting.

### Plasma analysis

Blood was collected from each hamster using heparinized capillary tubes and centrifugated to obtain plasma. Total cholesterol (TC) and triglyceride (TG) levels and LPL activity in the post-heparin plasma were assayed, and the lipid profiles of the lipoproteins were evaluated via the previous method [10].

### Hematoxylin and eosin staining

The embedded epididymal fat in Optimal Cutting Temperature Compound was cut and stained using the previous method [22].

### Real-time quantitative PCR (RT-qPCR)

Total RNAs were prepared via the Trizol method. The primers of the interested genes were listed in Table 1. RT-qPCR was performed as described in the previous study [18]. Relative gene expression was normalized to glycer-aldehyde-3-phosphate dehydrogenase (*GAPDH*) and calculated according to the 2^*-ΔΔCt*^ formula. *GAPDH* is a glycolytic enzyme and is widely used as an internal reference [23]. High-fat diet may alter the glycolytic activity, therefore, the gene expression in ezetimibe and CM1 groups were compared to the HFD group to reduce the potential effects of diet.

**Table 1:** The primers used for the polymerase chain reaction (PCR) reaction

Primer		Sequences (5′-3′)
GAPDH	Forward	CTCCCACTCTTCCACCTTTGATGC
hamster	Reverse	GTCCACCACTCTGTTGCTGTAGC
SREBP-2	Forward	GCTACTTCCTTAATCGTGCCCAGAG
hamster	Reverse	TCCTTGGCTGCTGACTTGATTGAC
SREBP-1c	Forward	GCGGACGCAGTCTGGG
hamster	Reverse	ATGAGCTGGAGCATGTCTTCAAA
ABCG8	Forward	TGCTGGCCATCATAGGGAG
hamster	Reverse	TCCTGATTTCATCTTGCCACC
LDLR	Forward	GCCGGGACTGGTCAGATG
hamster	Reverse	ACAGCCACCATTGTTGTCCA
LXRα	Forward	GCAGGACCAGCTCCAAGTAG
hamster	Reverse	GCAGGACCAGCTCCAAGTAG
PCSK9	Forward	TGCTCCAGAGGTCATCACAG
hamster	Reverse	GTCCCACTCTGTGACATGAAG
CYP7A1	Forward	GGTAGTGTGCTGTTGTATATGGGTTA
hamster	Reverse	ACAGCCCAGGTATGGAATCAAC
GAPDH	Forward	AGGTCGGTGTGAACGGATTTG
mouse	Reverse	GGGGTCGTTGATGGCAACA
PPARα	Forward	AACATCGAGTGTCGATATTGTGG
mouse	Reverse	CCGAATAGTTCGCCGAAAGAA
PPARγ	Forward	TCGCTGATGCACTGCCTATG
mouse	Reverse	GAGAGGTCCACAGAGCTGATT
FAS	Forward	CATCCACTCAGGTTCAGGTG
mouse	Reverse	AGGTATGCTCGCTTCTCTGC
ACC1	Forward	GAGGTACCGAAGTGGCATCC
mouse	Reverse	GTGACCTGAGCGTGGGAGAA
SCD1	Forward	CATCATTCTCATGGTCCTGCT
mouse	Reverse	CCCAGTCGTACACGTCATTTT
DGAT1	Forward	TGGTGTGTGGTGATGCTGATC
mouse	Reverse	GCCAGGCGCTTCTCAA
DGAT2	Forward	GGCTACGTTGGCTGGTAACT
mouse	Reverse	CACTCCCATTCTTGGAGAGC

### Immunoblotting analysis

This experiment was performed according to the previously reported methods [17–19]. For the epididymal fat samples, the lipids in the upper layer of the prepared homogenate were carefully removed after centrifugation. Immunoblotting was carried out using primary and secondary antibodies. Mouse monoclonal antibodies were used against PPARα (sc398394), β (sc-74,517) and γ (sc-7273), NPC1L1 (sc-166,802), adipose triglyceride lipase (ATGL, sc-365,278), sterol regulatory element binding protein (SREBP)-1c (sc-13,551) and − 2 (sc-271,616), apoAI (sc-58,230), and LPL (sc-373,759). A rabbit polyclonal antibody was used against LDLR (sc-18,823) (Santa Cruz, CA, USA). A rabbit polyclonal antibody was used against ATP-binding cassette (ABC) G8 (bs-10149R, Bioss, Beijing, China). A rabbit monoclonal antibody was used against scavenger receptor B type 1 (SR-B1, ab217318). A rabbit polyclonal antibody was used against liver X receptor (LXR) α (ab176323) (Abcam, MA, USA). Rabbit polyclonal antibodies were used against PCSK9 (55206–1-AP), albumin (16475–1-AP), and apoB (20578–1-AP) (Proteintech, IL, USA). A mouse monoclonal antibody was used against β-actin (66009–1-Ig) (Proteintech, IL, USA). A rabbit polyclonal antibody was used against cholesterol 7-α-hydroxylase A1 (CYP7A1, NP_000771) (OriGene, Shanghai, China).

### Data analysis

All the bioassay results were expressed as mean ± standard deviation (*SD*) for at least three independent experiments. One-way analysis of variance (ANOVA) analysis was used to detect significant difference between any two groups with SPSS19.0 software. Different letters were considered to be statistically significant at a *P* < 0.05, and the same letters meant there were no significant difference between two groups.

## Results

### CM1 intervention alleviated hyperlipidemia

**Fig. 2:**
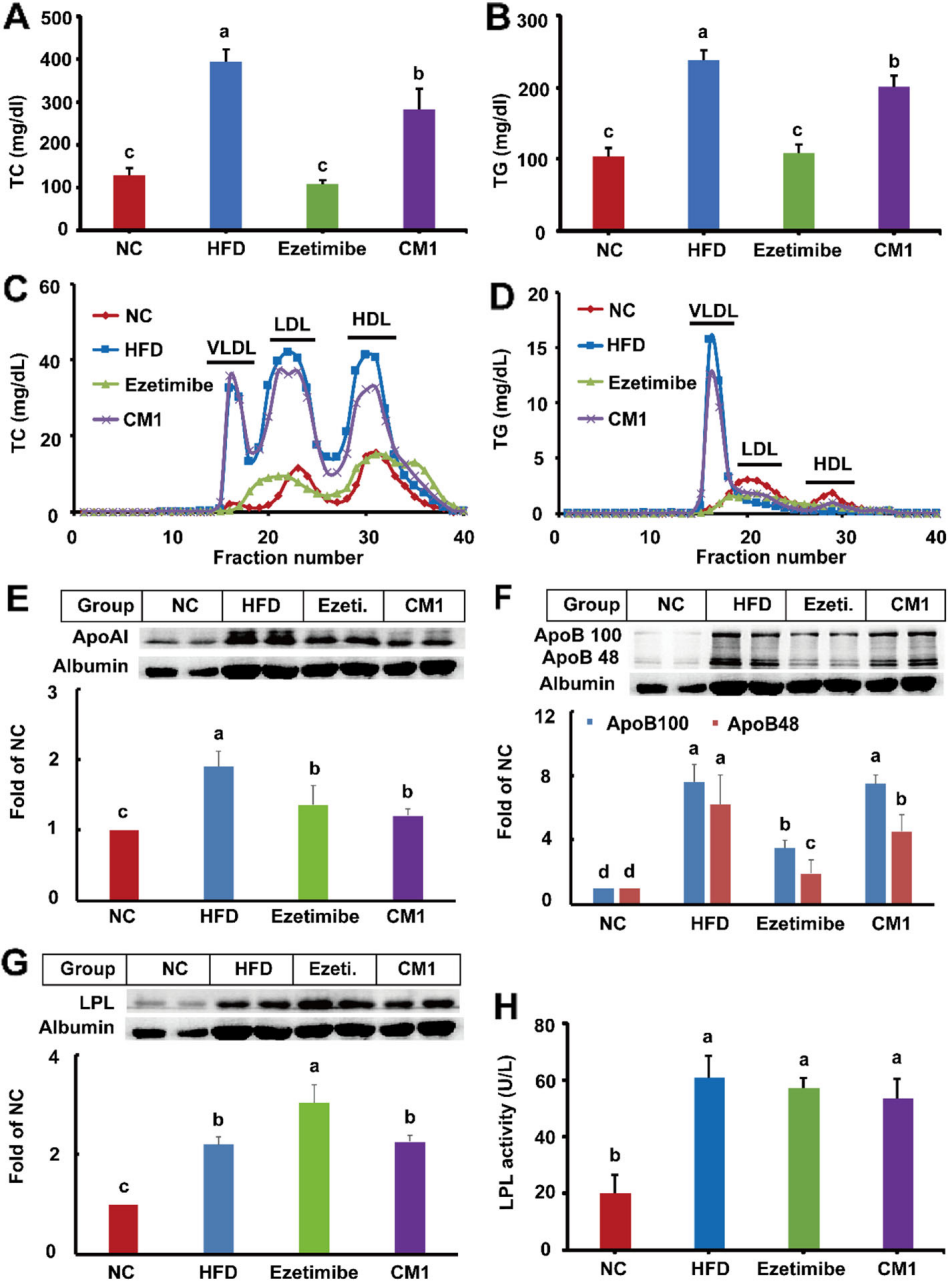
Effect of CM1 on the plasma lipid profiles and the expression of plasma apoAI, apoB and LPL (*n* = 5). A, plasma TC concentrations; B, plasma TG concentrations; C and D, plasma TC and TG profiles of the lipoproteins after ÄKTA-FPLC separation, respectively; E, F and G, plasma apoAI, apoB and LPL expression and densitometric quantification, respectively; H, plasma LPL activity. Data are presented as mean ± SD. Ezetimibe (Ezeti.): hamsters treated with ezetimibe at the dose of 25 mg/kg/d; CM1: LDLR^*(+/−)*^ hamsters treated with CM1 at the dose of 100 mg/kg/d. All the abbreviations applied for the rest of the figures

LDLR^*(+/−)*^ hamsters have an autosomal inherited hypercholesterolemia [15, 24]. The body weight of the hamsters in the NC, HFD, ezetimibe, and CM1 group increased approximately 29.1, 40.4, 41.0, and 32.1%, respectively, after feeding for 5 months (Fig. 1C, *P* < 0.01). Although the average food intake had no obvious differences among groups (Fig. 1D), CM1 intervention significantly decreased the final body weight of the hamsters (Fig. 1C, *P* < 0.05). Of note, HFD dramatically increased the average plasma TC (Fig. 2A, 128.8 vs 393.9 mg/dL) and TG (Fig. 2B, 104.6 vs 238.5 mg/dL) levels of the LDLR^*(+/−)*^ hamsters (*P* < 0.01). In line with previous studies [4, 24], ezetimibe also significantly decreased the elevated plasma TC level by ~ 72% and TG level by ~ 49% (*P* < 0.01). CM1 intervention notably decreased the plasma TC level by ~ 28% (Fig. 2A, *P* < 0.05, 283.2 vs 393.9 mg/dL) and TG level by ~ 16% (Fig. 2B, 201.2 vs 238.5 mg/dL). Furthermore, CM1 intervention significantly decreased the low-density lipoprotein (LDL) and high-density lipoprotein (HDL) cholesterol levels (Fig. 2C). It also reduced TG level in the very low-density lipoprotein (VLDL) fractions (Fig. 2D).

In line with HDL cholesterol, HFD dramatically enhanced the plasma apoAI level (Fig. 2E, *P* < 0.01). Of note, ezetimibe and CM1 intervention decreased the elevated plasma apoAI level by approximately 29% and ~ 36%, respectively (Fig. 2E, *P* < 0.05). Hamsters have both apoB100 and apoB48 in the plasma, which are mainly carried by non-HDL particles [15, 25, 26]. Ezetimibe reduced the elevated plasma apoB100 and apoB48 levels by approximately 54 and 69%, respectively (Fig. 2F, *P* < 0.01). Although CM1 intervention had no effect on the plasma level of apo100, this molecule significantly reduced the plasma level of apoB48 by 27% (Fig. 2F, *P* < 0.05). The alteration of plasma apoB48 was consistent with the plasma levels of TG. HFD also increased the plasma LPL level by 119% (Fig. 2G, *P* < 0.01). Of note, ezetimibe intervention significantly increased the level of plasma LPL protein by nearly 38% (*P* < 0.05). However, CM1 intervention did not affect the plasma level of LPL (Fig. 2G). In addition, HFD significantly increased plasma LPL activity (Fig. 2H, *P* < 0.01). In this study, neither ezetimibe nor CM1 intervention affected the plasma LPL activity (Fig. 2H).

### CM1 intervention modulated the liver genes

**Fig. 3:**
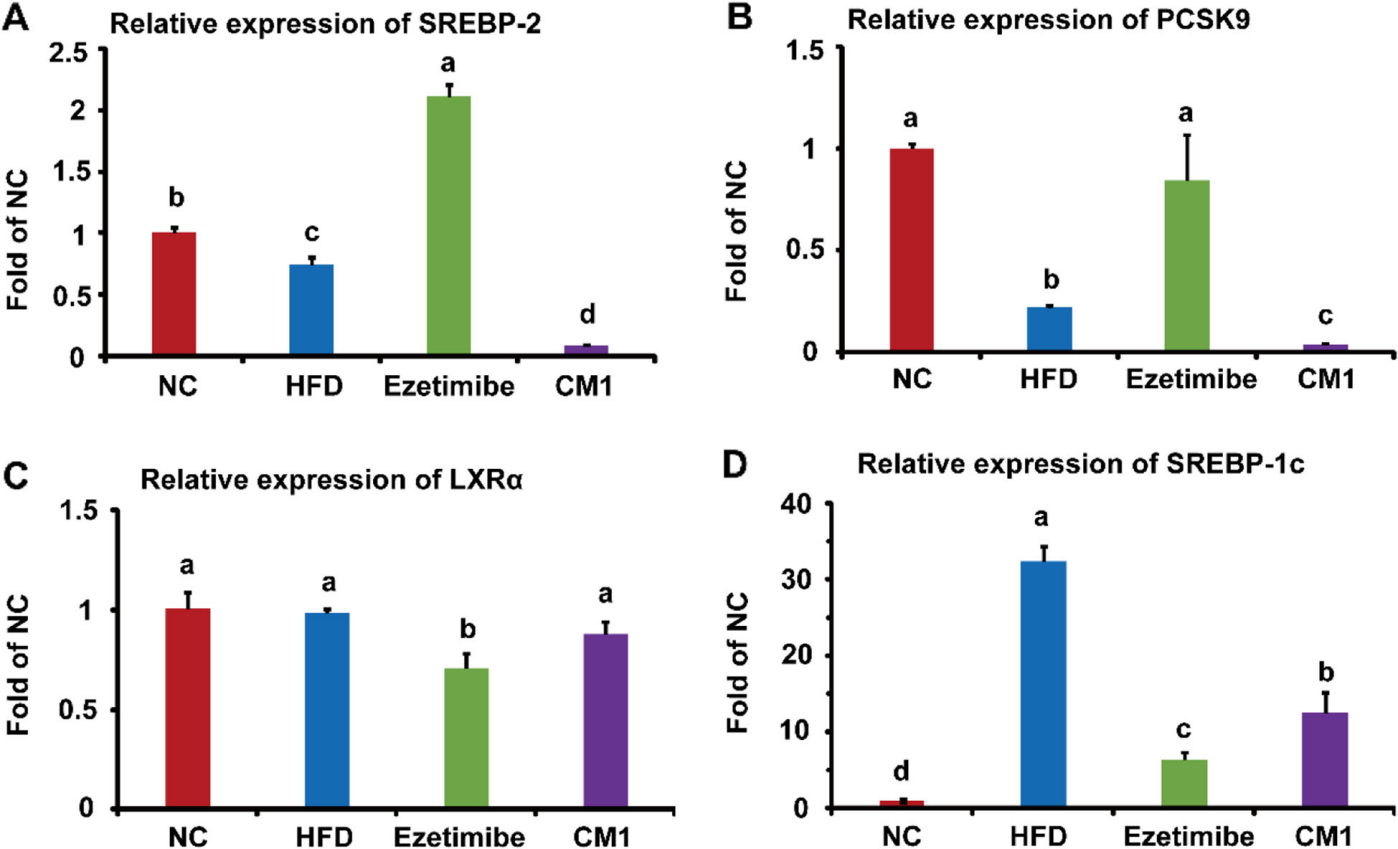
Effect of CM1 on the expression of lipid metabolism-related genes in the liver of LDLR^*(+/−)*^ hamsters (*n* = 3). A, B, C, and D, mRNA expression of SREBP-2, PCSK9, LXRα, and SREBP-1c, respectively

SREBP-2 modulate the expression of several genes, such as PCSK9 and LDLR, which are involved in cholesterol metabolism at the transcriptional level [16, 18, 27]. HFD dramatically reduced the gene expression of SREBP-2 and PCSK9 by approximately 26 and 78%, respectively (Fig. 3A and B, *P* < 0.01). Ezetimibe increased the gene expression of SREBP-2 by ~ 2.8-fold (Fig. 3A, *P* < 0.01) and PCSK9 by ~ 3.8-fold (Fig. 3B, *P* < 0.01) in comparison with the HFD group. Of note, CM1 intervention reduced the mRNA levels of SREBP-2 and PCSK9 by approximately 88 and 80%, respectively (Fig. 3A and B, *P* < 0.01). Furthermore, CM1 intervention also dramatically decreased the expression of these genes compared to the ezetimibe treatment (Fig. 3A and B, *P* < 0.01). Therefore, CM1 may decrease cholesterol synthesis at the transcriptional level.

LXRα is an important modulator of cholesterol metabolism [28]. HFD did not affect the gene expression of LXRα in this study (Fig. 3C). However, HFD dramatically increased the mRNA level of SREBP-1c by around 32-fold (Fig. 3D, *P* < 0.001). Ezetimibe treatment notably decreased the gene expression of LXRα by 28% (*P* < 0.05) and SREBP-1c by 80% (Fig. 3C and D, *P* < 0.01). CM1 also reduced the gene expression of SREBP-1c by approximately 61% (Fig. 3D, *P* < 0.05), but not LXRα (Fig. 3C). In this study, the C*t* numbers of LDLR, CYP7A1, and ABCG8 were greater than 30, suggesting the undetectable of these three genes.

### CM1 intervention improved the levels of CYP7A1 and ABCG5

**Fig. 4:**
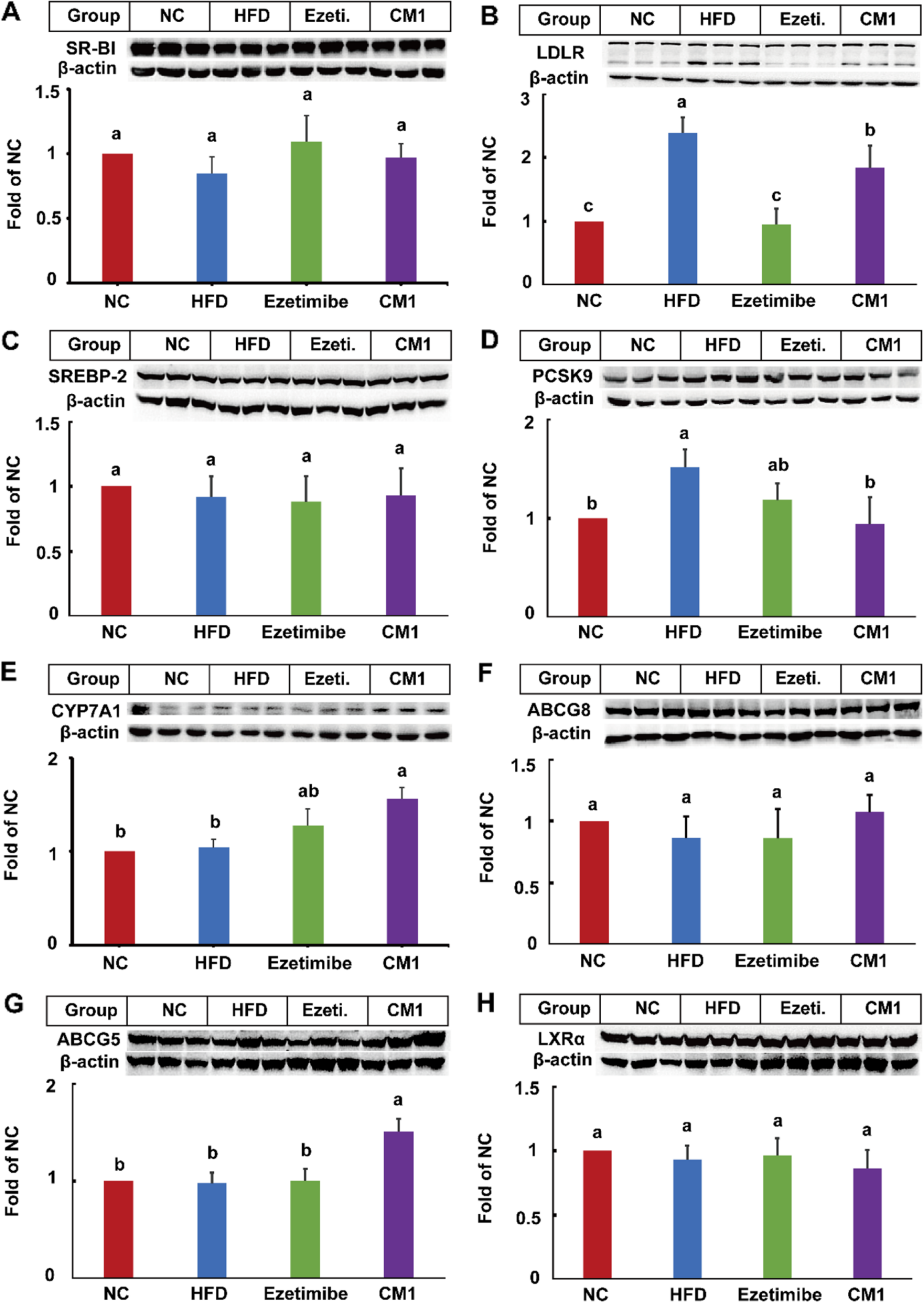
Effect of CM1 on the expression of TC metabolism-related proteins in the liver of the LDLR^*(+/−)*^ hamsters (*n* = 5). A, SR-B1; B, LDLR; C, SREBP-2; D, PCSK9; E, CYP7A1; F, ABCG8; G, ABCG5; H, LXRα expression and densitometric quantification

HFD notably increased the level of LDLR protein by 1.6-fold and PCSK9 protein by 53%, but not that of SR-BI and SREBP-2 (Fig. 4A-D). However, ezetimibe or CM1 administration had no effect on SR-B1 (Fig. 4A). Of note, ezetimibe decreased the protein expression of LDLR by 64% (Fig. 4B, *P* < 0.01). Compared to ezetimibe, CM1 notably increased the amount of LDLR protein (Fig. 4B, *P* < 0.05). As shown in Fig. 4D, CM1 dramatically decreased the expression of PCSK9 (38%, *P* < 0.05), a protein that can promote LDLR degradation [16, 29]. The changes of LDLR protein in ezetimibe and CM1 intervention groups were consistent with the alteration of PCSK9. Additionally, the expression of SREBP-2 had no significant difference in ezetimibe or CM1 intervention group (Fig. 4C).

**Fig. 5:**
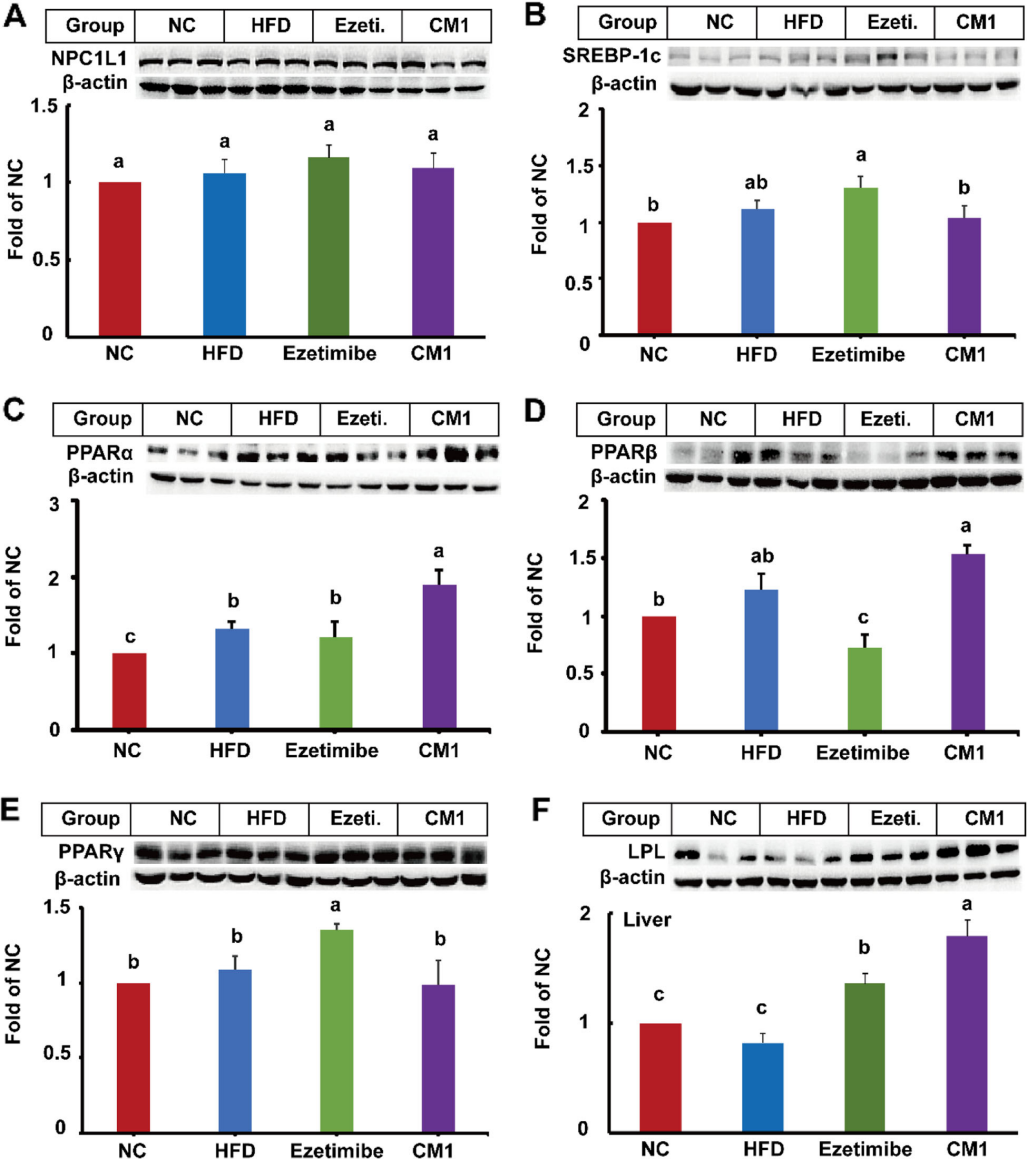
Effect of CM1 on the expression of NPC1L1 and TG metabolism-related proteins in the liver of the LDLR^*(+/−)*^ hamsters (*n* = 5). A, NPC1L1; B, SREBP-1c; C, PPARα; D, PPARβ; E, PPARγ; F, LPL expression and densitometric quantification

CYP7A1 is a key enzyme for bile acid synthesis [30]. CM1 treatment, but not ezetimibe, significantly enhanced the amount of CYP7A1 protein (Fig. 4E, *P* < 0.05). A proportion of cholesterol metabolites in the liver are transported to the gall bladder for excretion [28, 31]. In the present study, HFD and CM1 had no effect on the ABCG8 and LXRα proteins (Fig. 4F and H). However, CM1 treatment notably elevated the amount of hepatic ABCG5 protein in comparison with the HFD or ezetimibe treatment group (Fig. 4G, *P* < 0.05). In the liver, CM1 intervention did not affect the level of NPC1L1 protein (Fig. 5A), which mediates the reabsorption of biliary cholesterol [31, 32].

### CM1 intervention modulated TG metabolism-related proteins in the liver of LDLR^*(+/−)*^ hamsters

HFD enhanced the level of PPARα protein compared to the NC group (Fig. 5C, *P* < 0.05). Ezetimibe had no effect on SREBP-1c and PPARα proteins in comparison with the HFD group (Fig. 5B and C). However, ezetimibe decreased the expression of PPARβ by approximately 40% (Fig. 5D, *P* < 0.05) and increased the levels of PPARγ and LPL protein by 35 and 43%, respectively (Fig. 5E and F, *P* < 0.05). Of note, CM1 intervention enhanced the level of PPARα protein by approximately 43% (Fig. 5C, *P* < 0.05), but not that of SREBP-1c, PPARβ, or PPARγ (Fig. 5B, D and E). Furthermore, CM1 intervention notably enhanced the expression of PPARβ protein in comparison with the ezetimibe intervention (Fig. 5D, *P* < 0.01). Additionally, CM1 also increased the expression of LPL protein by 70% (Fig. 5F, *P* < 0.01) as that of ezetimibe.

### CM1 intervention inhibited the protein expression of NPC1L1 and SREBP-2 and enhanced the LXRα/ABCG8 in the gut

**Fig. 6:**
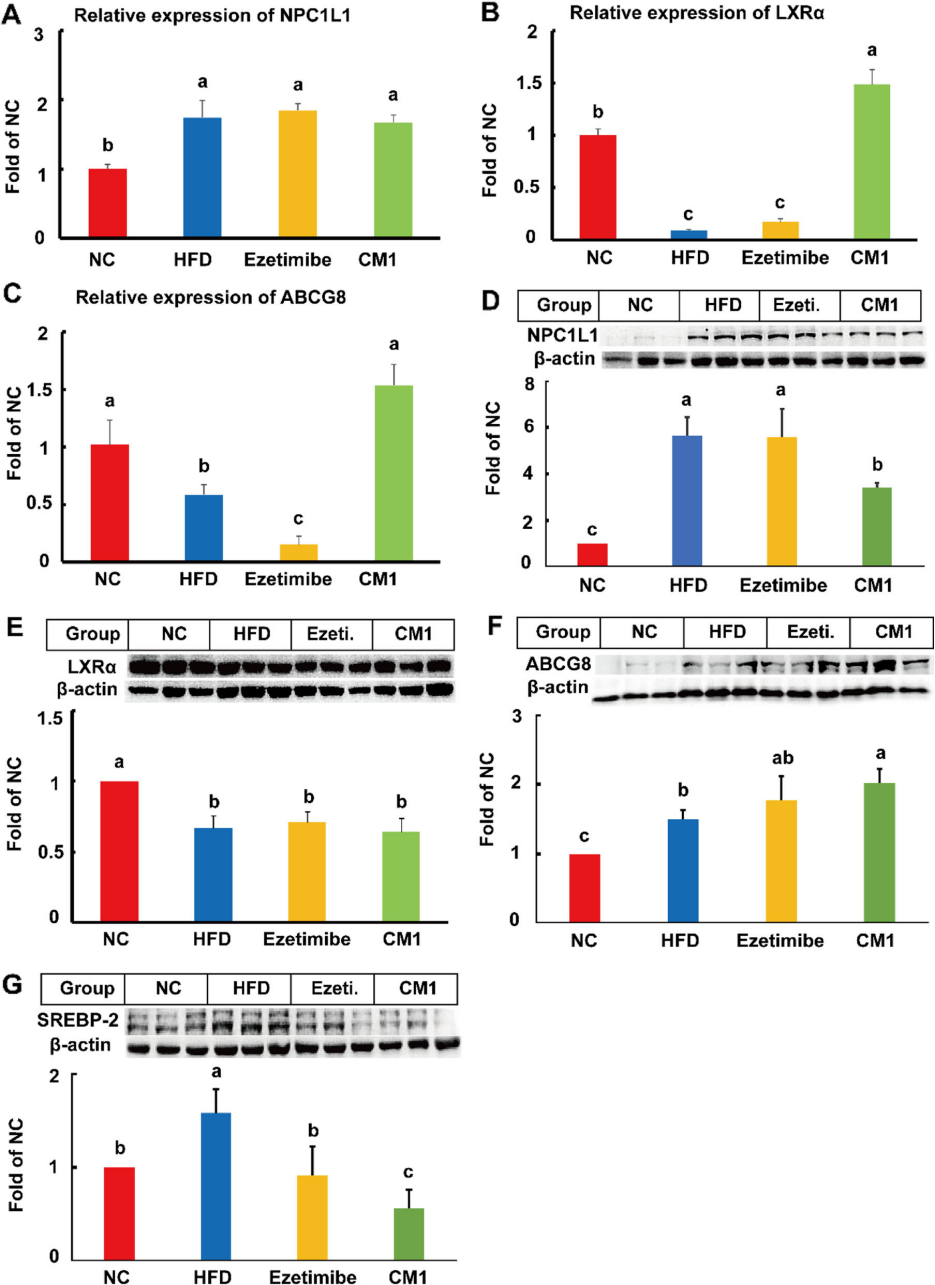
Effect of CM1 on the expression of lipid metabolism-related genes and proteins in the small intestine of the LDLR^*(+/−)*^ hamsters (*n* = 5 or 3). A, relative mRNA expression of NPC1L1; B, relative mRNA expression of LXRα; C, relative mRNA expression of ABCG8; D, NPC1L1; E, LXRα; F, ABCG8; G, SREBP-2 expression and densitometric quantification in the small intestine

In this study, HFD increased the mRNA expression of NPC1L1 by approximately 74% (Fig. 6A, *P* < 0.05). However, ezetimibe or CM1 intervention had no effect on the mRNA expression of NPC1L1. Furthermore, HFD notably reduced the mRNA expression of LXRα and ABCG8 by 92 and 41.5%, respectively (Fig. 6B and C). Ezetimibe reduced the mRNA level of ABCG8 by 73.8%, but not LXRα, compared to the high-fat diet group (Fig. 6 C). On the contrary, CM1 intervention increased the mRNA expression of LXRα by 15.8-fold and ABCG8 by 1.6-fold (Fig. 6B and C, *P* < 0.01) compared to the HFD group. In contrast to ezetimibe, CM1 intervention also enhanced the mRNA expression of LXRα and ABCG8 (*P* < 0.01, Fig. 6B and C). Additionally, SREBP-2 was undetectable in the small intestine due to the C*t* number was greater than 30.

Compared to the NC group, HFD increased the expression of NPC1L1 protein by approximately 4.6-fold (Fig. 6D, *P* < 0.01). Ezetimibe intervention showed no effect on the NPC1L1 protein in the present study. Mechanistically, ezetimibe prevents sterol-induced internalization of NPC1L1 [33, 34]. Of note, CM1 administration decreased the elevated NPC1L1 protein by around 39.5% (Fig. 6D, *P* < 0.05). HFD also increased the level of ABCG8 protein (*P* < 0.01) and decreased the LXRα protein (*P* < 0.05) in the small intestine (Fig. 6E and F). Compared to the HFD group, CM1 intervention dramatically increased the level of ABCG8 protein (Fig. 6F, *P* < 0.05), but not LXRα, in the small intestine. Furthermore, HFD intervention increased the level of SREBP-2 protein by 48% (*P* < 0.05) compared to the NC group (Fig. 6G). Ezetimibe significantly decreased the elevated SREBP-2 by 42% (Fig. 6G, *P* < 0.05). Similarly, CM1 intervention reduced the expression of SREBP-2 by 64% (Fig. 6G, *P* < 0.01) when compared with the HFD group. The inhibitory effect of CM1 on SREBP-2 was greater than that of ezetimibe (38% reduction, Fig. 6G, *P* < 0.05).

### CM1 modulated the lipid metabolism in the Epididymal fat

**Fig. 7:**
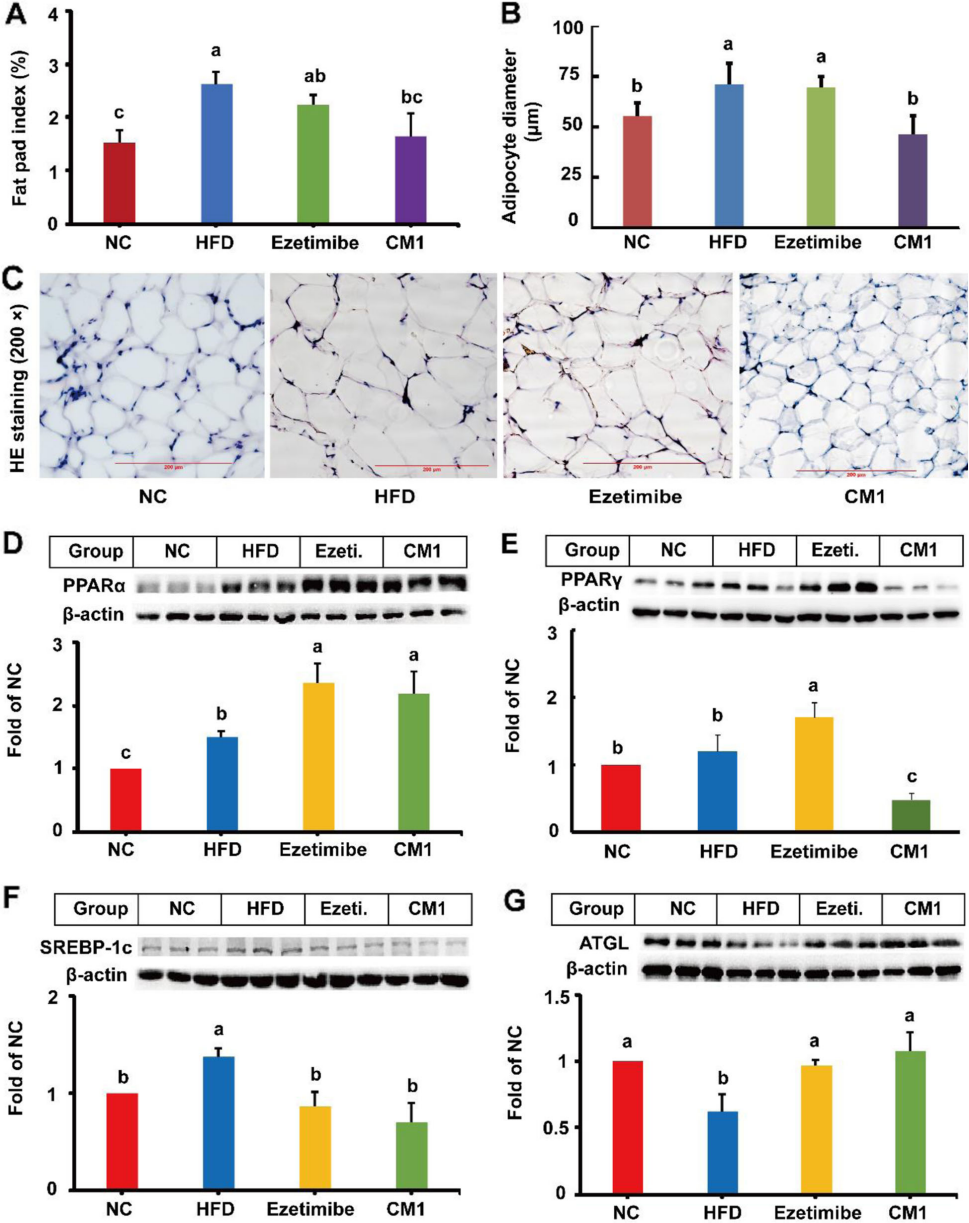
Effect of CM1 on the expression of lipid metabolism-related proteins in the epididymal fat of the LDLR^*(+/−)*^ hamsters (*n* = 5). A, fat pad index, and fat pad index means the percentage of fat pad to the body weight; B, adipocyte diameter in each group (*n* = 10); C, typical images of H & E staining (scale bar: 200 μm); D, PPARα; E, PPARγ; F, SREBP-1c; G, ATGL expression and densitometric quantification

In this study, HFD increased the fat pad index of the LDLR^*(+/−)*^ hamsters by approximately 74% (Fig. 7A, *P* < 0.01). CM1 administration, but not ezetimibe, significantly decreased the elevated fat pad index by around 39% (*P* < 0.05). HFD also increased the diameter of the adipocyte by 28.3% (*P* < 0.01), whereas CM1 treatment decreased the elevated diameter of the adipocyte by 34.9% (Fig. 7B and C, *P* < 0.01). Moreover, HFD increased the expression of PPARα and SREBP-1c proteins by approximately 48 and 38%, respectively, in the epididymal fat (Fig. 7D and F, *P* < 0.05). Of note, ezetimibe administration significantly decreased the expression of SREBP-1c (Fig. 7F) by 37%, and enhanced the expression of PPARα by 58% and PPARγ by 75% (Fig. 7D and E). Similarly, CM1 intervention reduced the expression of SREBP-1c by 49% (*P* < 0.05) and increased the level of PPARα by 46% (*P* < 0.05) when compared with the HFD group. Furthermore, CM1 intervention decreased the level of PPARγ protein by approximately 67% (Fig. 7E, *P* < 0.01). In the adipose tissue, ATGL promotes the hydrolysis of TGs and the production of fatty acids, thereby playing an important role in energy homeostasis [35]. As shown in Fig. 7G, HFD significantly decreased ATGL protein by approximately 38% (*P* < 0.01) compared to the regular chow diet. Ezetimibe or CM1 intervention enhanced the level of ATGL protein by 50 and 65%, respectively, compared to the HFD group (Fig. 7G, *P* < 0.05).

### CM1 decreased the lipid droplet formation in vitro

**Fig. 8:**
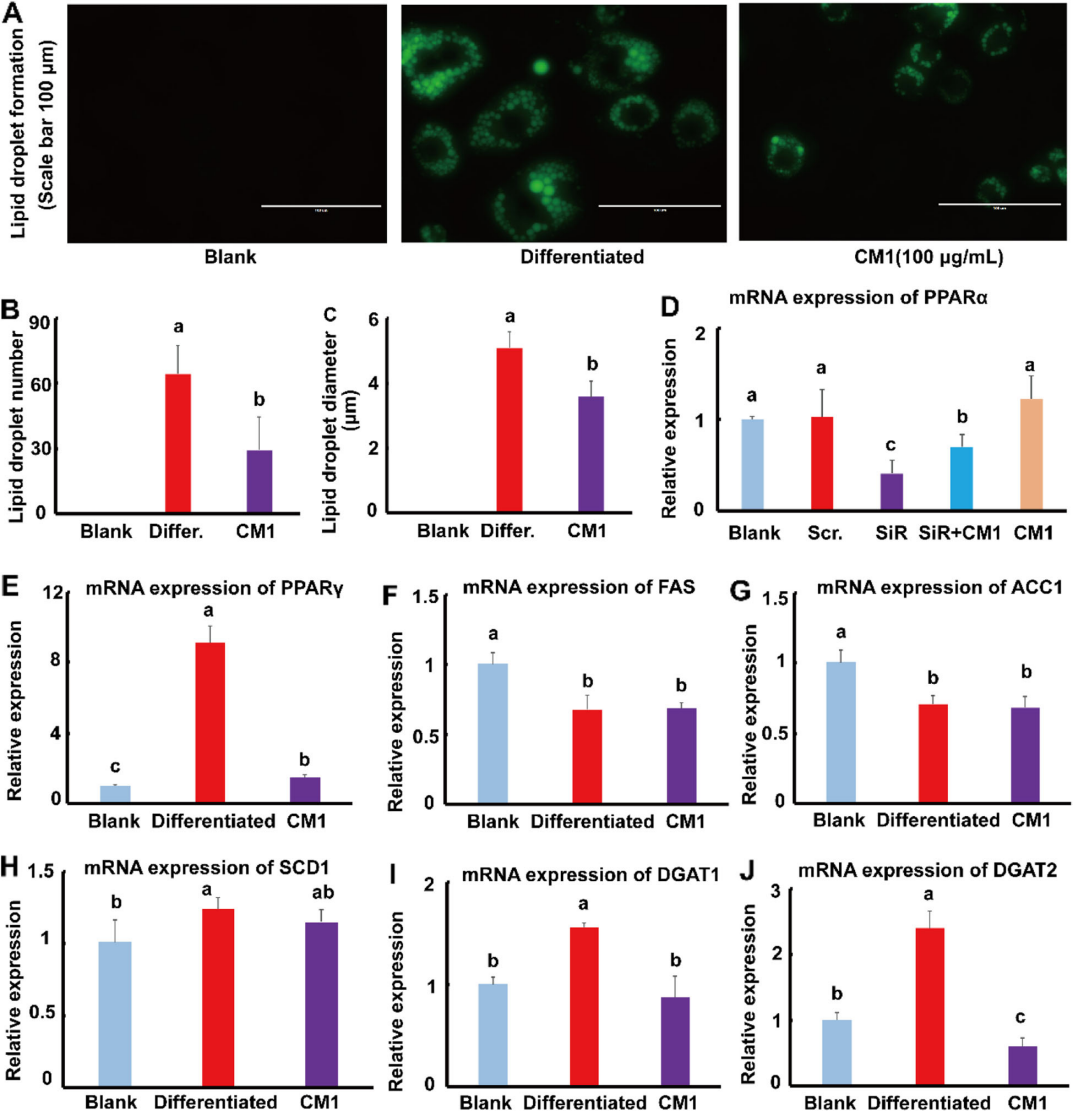
Effect of CM1 on the differentiation of 3T3-L1 cells induced by insulin. A, typical images of the lipid droplet in 3T3-L1 cells induced by insulin and the effect of CM1 at 100 μg/mL (scale bar: 100 μm); B, the average lipid droplet number in 3T3-L1 cells (*n* = 10); C, the average lipid droplet diameter in 3T3-L1 cells (*n* = 10); D, PPARα; E, PPARγ; F, FAS; G, ACC1; H, SCD1; I, DGAT1; and J, DGAT2 mRNA expression (*n* = 3). Differ.: 3T3-L1 cells were differentiated with chemical reagents as described in the method section

As shown in Fig. 8A, insulin successfully induced the formation of lipid droplet in 3T3-L1 cells, whereas CM1 intervention obviously decreased the lipid droplet formation. In this study, lipid droplet formation was not observed in the blank group. Therefore, the effect of CM1 intervention was only compared to the differentiated group. Statistically, CM1 intervention decreased the average number lipid droplets by 54.2% (*P* < 0.01). It also reduced the average diameter of lipid droplets by 29.7% (Fig. 8C, *P* < 0.01). In the differentiated group, the mRNA expression of PPARγ increased by 13.7-fold (Fig. 8E, *P* < 0.01). Furthermore, the levels of stearoyl-CoA desaturase 1 (SCD1), diacylglycerol acyltransferase (DGAT) 1 and 2 enhanced 1.4-fold (*P* < 0.01), 55.8% (*P* < 0.05), and 1.4-fold (*P* < 0.01), respectively, compared with the blank group (Fig. 8H, I, and J). PPARα siRNA dramatically reduced the mRNA expression of PPARα by approximately 59% compared to the scrambled siRNA group (*P* < 0.05), while CM1 intervention increased the mRNA expression of PPARα by approximately 72% compared to the single PPARα siRNA treatment group (*P* < 0.05, Fig. 8D). The gene expression of fatty acid synthase (FAS) and acetyl-CoA carboxylase 1 (ACC1) decreased by approximately 29% in the differentiated group compared to the blank group (Fig. 8F and G, *P* < 0.05). The above results further demonstrated that PPARγ is important for adipocyte differentiation. It is worth noted that CM1 intervention decreased the mRNA expression of PPARγ, DGAT1, and DGAT2 by 83.8% (Fig. 8E, *P* < 0.01), 43.8% (Fig. 8I, *P* < 0.05), and 74.7% (Fig. 8J, *P* < 0.01), respectively. Furthermore, CM1 intervention did not affect the mRNA expression of PPARα, SCD1, FAS, and ACC1 when compared with the differentiated group.

## Discussion

The water extracts or polysaccharides of the *Cordyces* species have anti-hyperlipidemic effects in mice [10–13]. However, the lipid profile in mice is distinct from humans [25]. In this study, the polysaccharide CM1 from *C. militaris* displayed powerful hypolipidemic effects in LDLR^*(+/−)*^ hamsters, which have a human-like lipid profile [4, 15, 24]. When compared with ezetimibe treatment, CM1 showed distinct effects on many genes and proteins. Therefore, CM1 exerts hyperlipidemic effects by distinct mechanisms compared to ezetimibe as a result of structural differences (Fig. 1A).

Firstly, CM1 may reduce plasma TC by enhancing reverse cholesterol transport. SR-BI and LDLR mediate cholesterol transport from lipoproteins to the liver, contributing to a reduction of plasma TC [16, 27, 36]. A recent study suggested that CM1 can enhance the expression of SR-B1 protein in apoE^*(−/−)*^ mice [37]. However, CM1 had no effect on SR-BI protein in this study, and it reduced the plasma level of apoAI, an important acceptor of peripheral cholesterol [17, 38], as that of ezetimibe. Although the plasma apoAI level was similar, CM1 intervention seemed to enhance the level of HDL cholesterol compared to ezetimibe, suggesting CM1 may improve the transfer of plasma cholesterol from HDL particles to the liver. A previous study already demonstrated that CM1 may improve HDL-mediated cholesterol efflux [17]. Recent studies also indicated that water-soluble components of *Cordyceps* species can lower hyperlipidemia [10–13, 39]. This study suggested that CM1 is one of the water-soluble components with hypolipidemic effects.

The potential LDLR promoting effect of CM1 was consistent with the finding in apoE^*(−/−)*^ mice [37]. Of note, the reductions of PCSK9 at the gene and protein levels in CM1 group were consistent with the decrease of SREBP-2 at the transcription level. However, a recent study demonstrated that the polysaccharide CM3-SII can enhance the SREBP-2 pathway in Huh7 cells [19]. These differences may be caused by either the distinct structure of the polysaccharides or the experimental models. In this study, although ezetimibe enhanced the gene expression of SREBP-2 and PCSK9, it significantly reduced the level of LDLR protein. These data suggested that ezetimibe may modulate these proteins at a post-transcriptional level. Additionally, the inconsistency of the effect of ezetimibe on the SREBP-2 pathway between this study and previous studies may be attributed to the short intervention time (only 3 or 7 days) and the regular chow diet rather than the high-fat diet used in the previous studies [40–42].

Furthermore, CM1 intervention increased the protein expression of CYP7A1, which suggested that CM1 could improve the synthesis of bile acid. As found in the previous study [37], CM1 intervention may increase lipid excretion via enhancing the protein expression of ABCG5/8. Furthermore, the *C. militaris* polysaccharide CM3II could also enhance the LXRα/ABCG8 pathway in apoE^*(−/−)*^ mice [10]. Unlike the effect of ezetimibe, CM1 can directly inhibit the protein level of NPC1L1. Therefore, the polysaccharide CM1 may inhibit NPC1L1-mediated cholesterol absorption from small intestine by a distinct mechanism compared to ezetimibe. These data suggested that the TC-lowering effect of CM1 may be, at least in part, attributed to the reduction of NPC1L1-mediated cholesterol absorption, SR-B1-mediated cholesterol uptake, CYP7A1-mediated cholesterol conversion, and the subsequent ABCG5/8-mediated lipid excretion.

Secondly, CM1 may reduce plasma TG concentration by inhibiting TG synthesis and promoting fatty acid degradation. Similar as that of ezetimibe, CM1 may reduce TG synthesis by inhibiting the transcription of SREBP-1c. Accumulating evidence have demonstrated that SREBP-1c can regulate the expression of lipogenic genes at the transcriptional level, thereby modulating TG metabolism [18, 27]. Unlike ezetimibe, CM1 had no effect on the plasma level of apoB100. However, CM1 reduced the plasma level of apoB48 as that of ezetimibe, suggesting this molecule may act on the apoB48 editing in the small intestine rather than apoB100 editing in the liver. The effect of ezetimibe on apoB and VLDL particles were consistent with previous studies [43–45]. Mechanistically, ezetimibe can inhibit apoB secretion and reduce the production and export of chylomicron and VLDL particles [43, 44, 46]. A reduction of TG level in the plasma VLDL fractions of CM1-treated hamsters was observed compared to the HFD group. This reduction can be partially attributed to the reduced apoB48 level but no the plasma LPL, a key enzyme in hydrolysis of lipoprotein and chylomicron [47, 48]. Furthermore, PPARs are activated by a large variety of fatty acids and their derivatives. PPARα and PPARβ are major inducers of fatty acid oxidation in liver, whereas PPARγ is a major activator of adipocyte differentiation [37, 49]. This study suggested that CM1 can lower TG by upregulating PPARα-mediated β-oxidation and LPL-mediated lipoprotein degradation in the liver. A recent integrated bioinformatics analysis demonstrated that CM1 can regulate the PPAR signaling in apoE^*(−/−)*^ mice [37]. From the perspective of structure-activity relationship, the modulatory effects of CM1 on PPARα and SREBPs may be attributed to the β-D-linked glycosyls contained in CM1 [10, 50]. It has previously been shown that *Ganoderma lucidum* polysaccharides containing β-D-glucans can inhibit fatty acid synthesis by inhibiting SREBP-1c [51, 52].

Thirdly, CM1 may inhibit adipocyte differentiation in LDLR^*(+/−)*^ hamsters. A previous study demonstrated that the fermented *C. militaris* extract can inhibit adipocyte hypertrophy in mice [13]. As PPARγ is a major activator of adipocyte differentiation [49], the inhibitory effect of CM1 on the adipose of the LDLR^*(+/−)*^ hamster can be partially attributed to its downregulation of PPARγ. Other natural polysaccharides, such as fucoidan, also have the ability of inhibiting PPARγ [53]. Furthermore, the enhanced protein expression of PPARα in the adipose may increase the degradation of fatty acids, contributing to the reduced fat pad index. Additionally, the reduction of adipose in the CM1 treatment group may also be attributed to the enhanced expression of ATGL, which can promote the hydrolysis of TGs and the production of fatty acids [35]. In line with the results seen in the adipose, the bioactive polysaccharide CM1 can inhibit the differentiation of 3T3-L1 cells by downregulation of PPARγ. Furthermore, CM1 may inhibit TG synthesis via reducing DGAT1 and DGAT2 [54], thereby inhibiting adipocyte differentiation.

### Strengths and limitations

**Fig. 9:**
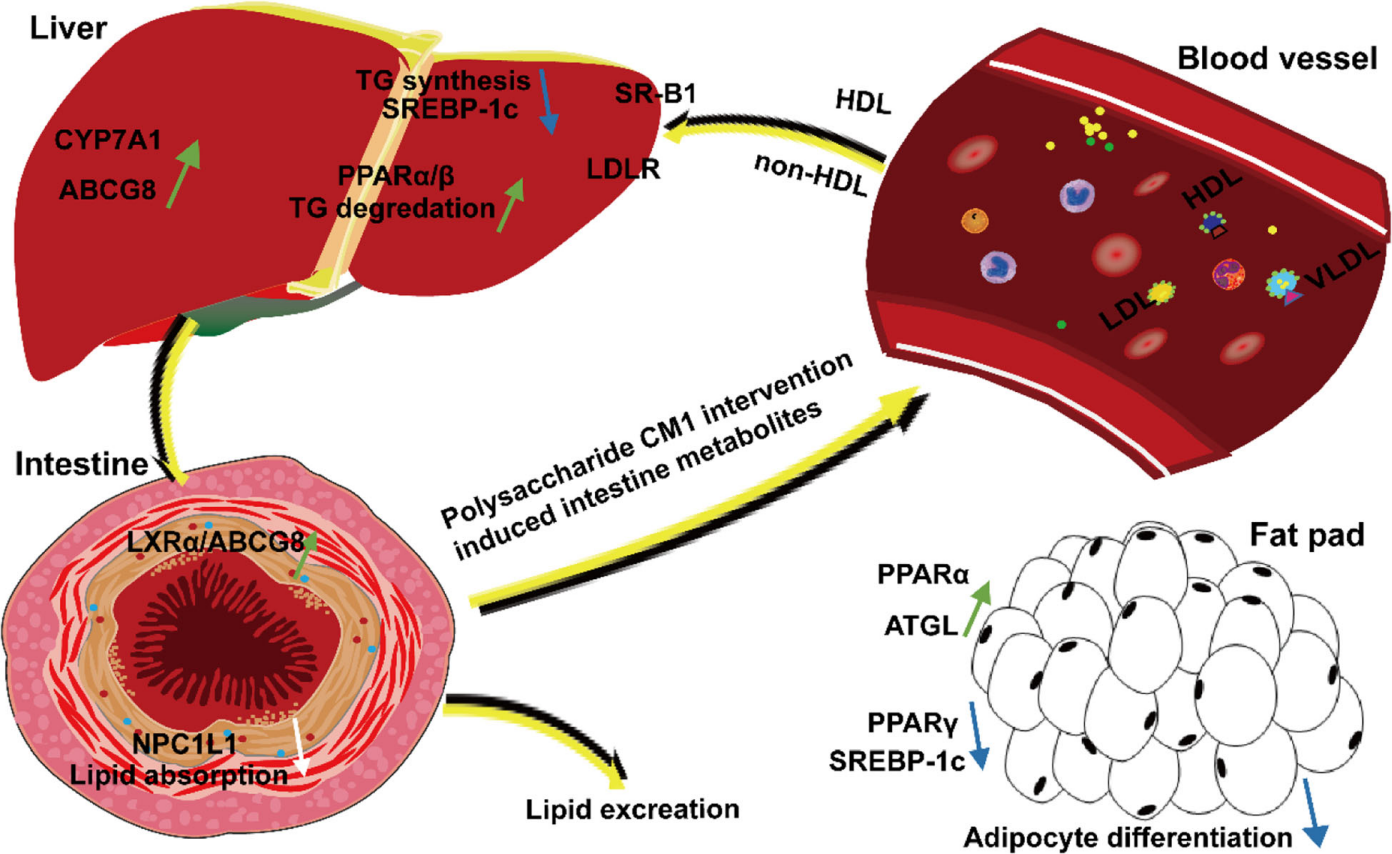
The proposed mechanisms of action of the polysaccharide CM1 in LDLR^*(+/−)*^ hamsters. We proposed that the polysaccharide CM1 or its physiologically degraded products may directly act on the gut system. Alternatively, the changed gut metabolites that induced by CM1 or even the degraded products may finally reach the circulation to exert their functions

As concluded in Fig. 9, this bioactive polysaccharide CM1 can alleviate hyperlipidemia and adipocyte differentiation in LDLR^*(+/−)*^ hamsters by several proposed mechanisms. Firstly, it increases the levels of CYP7A1 and ABCG5/8, that may contribute to the potential conversion and excretion of cholesterol, respectively. Secondly, CM1 decreases the protein expression of NPC1L1 and SREBP-2 in the gut, which may lead to a potential reduction of cholesterol absorption and synthesis. Thirdly, it may lower TG via enhancing the levels of LPL and PPARα in the liver and decreasing the apoB48 production in the small intestine. Finally, CM1 intervention leads to a reduction of adipocyte differentiation potentially by modulating multiple molecules in the epididymal fat.

The limitations of this study are listed below. (1), as CM1 reduced the expression of NPC1L1 protein, whether this polysaccharide can inhibit intestinal cholesterol absorption is an interesting question and should be answered in future studies. (2), genes exert their functions after being translated to the corresponding proteins, and many factors may influence the translational process, contributing to the inconsistent of the gene and protein expression. As a limitation of this study, whether CM1 affects the above process need to be clarified in the future. (3), as a primary study, only some genes related to adipocyte differentiation were examined in vitro. Whether CM1 can influence the expression of proteins that associated with adipocyte differentiation need to be investigated by comparation with a suitable positive control, such as PPARγ agonist, which has been demonstrated to act on adipocyte differentiation. Importantly, polysaccharide may be degraded in vivo by gastric acid and/or gut microbiota, it is impelled to investigate the effect and especially the mechanisms of CM1 in vitro using its physiologically degraded products. (4), given the big molecular weight of the polysaccharide CM1, its action both in vitro and in vivo is an interesting topic to be studied in the future. One possible mechanism by which CM1 or its physiologically degraded products modulate these genes or proteins in different organs might be by affecting gut microbiota and the metabolites, that have been demonstrated to be associated with cardiovascular diseases [55, 56]. Future research is needed to clarify what effect CM1 has on gut microbiota, and how other potential effects contribute to CM1-mediated lipid homeostasis.

## Conclusions

This long-term study demonstrated for the first time that the polysaccharide CM1 from the fruiting body of *C. militaris* has an attractive effect on lowering hyperlipidemia in LDLR^*(+/−)*^ hamsters via influencing on multiple pathways. These findings provide evidence that the polysaccharide CM1 can be used for treatment of the patients with abnormal lipid profiles as monotherapy or in combination with other lipid-lowering compounds. Alternatively, CM1 can be supplemented as a food additive for daily care of the patients with hyperlipidemia. Therefore, this study highlights the potential applications of polysaccharides from *C. militaris* in both food and pharmaceutical areas.

## Supplementary Information

**Additional file 1.**

## Data Availability

The datasets used and/or analyzed during the current study are available from the corresponding author on reasonable request.

## Abbreviations

ABC: ATP binding cassette
ACC1: Acetyl-CoA carboxylase 1
Apo: Apolipoprotein
ATGL: Adipose triglyceride lipase
CETP: Cholesteryl ester transfer protein
CVD: Cardiovascular disease
CYP7A1: Cholesterol 7-α-hydroxylase A1
DGAT: Diacylglycerol acyltransferase
FAS: Fatty acid synthase
HDL: High-density lipoprotein
LDLR: Low-density lipoprotein receptor
LDLR^*(−/−)*^: Low-density lipoprotein receptor (LDLR)-deficient
LDLR^*(+/−)*^: Heterozygous LDLR-deficient
LPL: Lipoprotein lipase
LXR: Liver X receptor
NPC1L1: Niemann-Pick C1-like 1
PPAR: Peroxisome proliferator-activated receptor
PCSK9: Proprotein convertase subtilisin/kexin type 9
RT-qPCR: Real-time quantitative PCR
SCD1: Stearoyl-CoA desaturase 1
SR-B1: Scavenger receptor B type 1
SREBP: Sterol regulatory element-binding protein
TC: Total cholesterol
TG: Triglyceride
VLDL: Very low-density lipoprotein

## References

[1] Zhao D, Liu J, Wang M, Zhang X, Zhou M (2019). Epidemiology of cardiovascular disease in China: current features and implications. Nat Rev Cardiol.

[2] Leong DP, Joseph PG, McKee M, Anand SS, Teo KK, Schwalm JD, Yusuf S (2017). Reducing the global burden of cardiovascular disease, part 2: prevention and treatment of cardiovascular disease. Circ Res.

[3] Soppert J, Lehrke M, Marx N, Jankowski J, Noels H (2020). Lipoproteins and lipids in cardiovascular disease: from mechanistic insights to therapeutic targeting. Adv Drug Deliv Rev.

[4] Xia B, Lin P, Ji Y, Yin J, Wang J, Yang X, Li T, Yang Z, Li F, Guo S (2020). Ezetimibe promotes CYP7A1 and modulates PPARs as a compensatory mechanism in LDL receptor-deficient hamsters. Lipids Health Dis.

[5] Wang DQ (2007). Regulation of intestinal cholesterol absorption. Annu Rev Physiol.

[6] Garcia-Calvo M, Lisnock J, Bull HG, Hawes BE, Burnett DA, Braun MP, Crona JH, Davis HR, Dean DC, Detmers PA, Graziano MP, Hughes M, Euan Macintyre D, Ogawa A, O'neill KA, Iyer SPN, Shevell DE, Smith MM, Tang YS, Makarewicz AM, Ujjainwalla F, Altmann SW, Chapman KT, Thornberry NA (2005). The target of ezetimibe is Niemann-pick C1-like 1 (NPC1L1). Proc Natl Acad Sci U S A.

[7] Panda AK, Swain KC (2011). Traditional uses and medicinal potential of *Cordyceps sinensis* of Sikkim. J Ayurveda Integr Med.

[8] Zhang J, Wen C, Duan Y, Zhang H, Ma H (2019). Advance in *Cordyceps militaris* (Linn) link polysaccharides: isolation, structure, and bioactivities: a review. Int J Biol Macromol.

[9] Cui JD (2015). Biotechnological production and applications of *Cordyceps militaris*, a valued traditional Chinese medicine. Crit Rev Biotechnol.

[10] Yang X, Lin P, Wang J, Liu N, Yin F, Shen N, Guo S (2021). Purification, characterization and anti-atherosclerotic effects of the polysaccharides from the fruiting body of *Cordyceps militaris*. Int J Biol Macromol.

[11] Koh JH, Kim JM, Chang UJ, Suh HJ (2003). Hypocholesterolemic effect of hot-water extract from mycelia of *Cordyceps sinensis*. Biol Pharm Bull.

[12] Kim SB, Ahn B, Kim M, Ji HJ, Shin SK, Hong IP, Kim CY, Hwang BY, Lee MK (2014). Effect of *Cordyces militaris* extract and active constituents on metabolic parameters of obesity induced by high-fat diet in C58BL/6J mice. J Ethnopharmacol.

[13] Tran NKS, Kim GT, Park SH, Lee D, Shim SM, Park TS (2019). Fermented *Cordyceps militaris* extract prevents hepatosteatosis and adipocyte hypertrophy in high fat diet-fed mice. Nutrients.

[14] Paterson RR (2008). Cordyceps: a traditional Chinese medicine and another fungal therapeutic biofactory?. Phytochemistry.

[15] Guo X, Gao M, Wang Y, Lin X, Yang L, Cong N, An X, Wang F, Qu K, Yu L, Wang Y, Wang J, Zhu H, Xian X, Liu G (2018). LDL receptor gene-ablated hamsters: a rodent model of familial hypercholesterolemia with dominant inheritance and diet-induced coronary atherosclerosis. EBioMedicine.

[16] Guo S, Xia XD, Gu HM, Zhang DW (2020). Proprotein convertase subtilisin/Kexin-type 9 and lipid metabolism. Adv Exp Med Biol.

[17] Hu S, Wang J, Li F, Hou P, Yin J, Yang Z, Yang X, Li T, Xia B, Zhou G, Liu M, Song W, Guo S (2019). Structural characterisation and cholesterol efflux improving capacity of the novel polysaccharides from *Cordyceps militaris*. Int J Biol Macromol.

[18] Li T, Hu SM, Pang XY, Wang JF, Yin JY, Li FH, Wang J, Yang XQ, Xia B, Liu YH, Song WG, Guo SD (2020). The marine-derived furanone reduces intracellular lipid accumulation in vitro by targeting LXRα and PPARα. J Cell Mol Med.

[19] Wang J, Wang Y, Yang X, Lin P, Liu N, Li X, Zhang B, Guo S (2021). Purification, structural characterization, and PCSK9 secretion inhibitory effect of the novel alkali-extracted polysaccharide from *Cordyceps militaris*. Int J Biol Macromol.

[20] Adomshick V, Pu Y, Veiga-Lopez A (2020). Automated lipid droplet quantification system for phenotypic analysis of adipocytes using CellProfiler. Toxicol Mech Methods.

[21] Guo SD, Cui YJ, Wang RZ, Wang RY, Wu WX, Ma T (2014). Separation, purification and primary reverse cholesterol transport study of *Cordyceps militaris* polysaccharide. China J Chin Mater Med.

[22] Yin J, Wang J, Li F, Yang Z, Yang X, Sun W, Xia B, Li T, Song W, Guo S (2019). The fucoidan from the brown seaweed *Ascophyllum nodosum* ameliorates atherosclerosis in apolipoprotein E-deficient mice. Food Funct.

[23] Zhang JY, Zhang F, Hong CQ, Giuliano AE, Cui XJ, Zhou GJ, Zhang GJ, Cui YK (2015). Critical protein GAPDH and its regulatory mechanisms in cancer cells. Cancer Biol Med.

[24] Wu Y, Xu MJ, Cao Z, Yang C, Wang J, Wang B, Liu J, Wang Y, Xian X, Zhang F, Liu G, Chen X (2019). Heterozygous Ldlr-deficient hamster as a model to evaluate the efficacy of PCSK9 antibody in hyperlipidemia and atherosclerosis. Int J Mol Sci.

[25] Gao S, He L, Ding Y, Liu G (2010). Mechanisms underlying different responses of plasma triglyceride to high-fat diets in hamsters and mice: roles of hepatic MTP and triglyceride secretion. Biochem Biophy Res Commun.

[26] Reaves SK, Wu JY, Wu Y, Fanzo JC, Wang YR, Lei PP, Lei KY (2000). Regulation of intestinal apolipoprotein B mRNA editing levels by a zinc-deficient diet and cDNA cloning of editing protein in hamsters. J Nutr.

[27] Moslehi A, Hamidi-Zad Z (2018). Role of SREBPs in liver diseases: a mini-review. J Clin Transl Hepatol.

[28] Guo S, Li L, Yin H (2018). Cholesterol homeostasis and liver X receptor (LXR) in atherosclerosis. Cardiovasc Hematol Disord Drug Targets.

[29] Yang HX, Zhang M, Long SY, Tuo QH, Tian Y, Chen JX, Zhang CP, Liao DF (2020). Cholesterol in LDL receptor recycling and degradation. Clin Chim Acta.

[30] Ge MX, Shao RG, He HW (2019). Advances in understanding the regulatory mechanism of cholesterol 7α-hydroxylase. Biochem Pharmacol.

[31] Kidambi S, Patel SB (2008). Cholesterol and non-cholesterol transporters: ABCG5, ABCG8 and NPC1L1: a review. Xenobiotica.

[32] Pramfalk C, Jiang ZY, Parini P (2011). Hepatic Niemann-pick C1-like 1. Curr Opin Lipidol.

[33] Davis HR, Veltri EP (2007). Zetia: inhibition of Niemann-pick C1 like 1 (NPC1L1) to reduce intestinal cholesterol absorption and treat hyperlipidemia. J Atheroscler Tromb.

[34] Ge L, Wang J, Qi W, Miao HH, Cao J, Qu YX, Li BL, Song BL (2008). The cholesterol absorption inhibitor ezetimibe acts by blocking the sterol-induced internalization of NPC1L1. Cell Metab.

[35] Schreiber R, Xie H, Schweiger M (2019). Of mice and men: the physiological role of adipose triglyceride lipase (ATGL). Biochim. Biophys. Acta Mol Cell Biol Lipids.

[36] Ma B, Jia J, Wang X, Zhang R, Niu S, Ni L, Di X, Liu C (2020). Differential roles of scavenger receptor class B type I: a protective molecule and a facilitator of atherosclerosis (review). Mol Med Rep.

[37] Lin P, Yin F, Shen N, Liu N, Zhang B, Li Y, et al. Integrated bioinformatics analysis of the anti-atherosclerotic mechanisms of the polysaccharide CM1 from Cordyceps militaris. Int J Biol Macromol. 2021. 10.1016/j.ijbiomac.2021.10.175. 34757129

[38] Cucuianu M, Coca M, Hâncu N (2007). Reverse cholesterol transport and atherosclerosis. A mini review. Rom. J Intern Med.

[39] Yamaguchi Y, Kagota S, Nakamura K, Shinozuka K, Kunitomo M. Inhibitory effects of water extracts from fruiting bodies of cultured *Cordyceps sinensis* on raised serum lipid peroxide levels and aortic cholesterol deposition in atherosclerotic mice. Phytother Res. 2000;14(8):650–2. https://doi.org/10.1002/1099-1573(200012)14:8<650::AID-PTR675>3.0.CO;2-0. 10.1002/1099-1573(200012)14:8<650::aid-ptr675>3.0.co;2-0 11114007

[40] Catry E, Pachikian BD, Salazar N, Neyrinck AM, Cani PD, Delzenne NM (2015). Ezetimibe and simvastatin modulate gut microbiota and expression of genes related to cholesterol metabolism. Life Sci.

[41] Engelking LJ, McFarlane MR, Li CK, Liang G (2012). Blockade of cholesterol absorption by ezetimibe reveals a complex homeostatic network in enterocytes. J Lipid Res.

[42] Xu RX, Liu J, Li XL, Li S, Zhang Y, Jia YJ, Sun J, Li JJ (2015). Impacts of ezetimibe on PCSK9 in rats: study on the expression in different organs and the potential mechanisms. J Transl Med.

[43] Nakano T, Inoue I, Takenaka Y, Ito R, Kotani N, Sato S, Nakano Y, Hirasaki M, Shimada A, Murakoshi T (2020). Ezetimibe impairs transcellular lipid trafficking and induces large lipid droplet formation in intestinal absorptive epithelial cells. Biochim. Biophys. Acta Mol Cell Biol Lipids.

[44] Telford DE, Sutherland BG, Edwards JY, Andrews JD, Barrett PH, Huff MW (2007). The molecular mechanisms underlying the reduction of LDL apoB-100 by ezetimibe plus simvastatin. J Lipid Res.

[45] Naples M, Baker C, Lino M, Iqbal J, Hussain MM, Adeli K (2012). Ezetimibe ameliorates intestinal chylomicron overproduction and improves glucose tolerance in a diet-induced hamster model of insulin resistance. Am J Physiol Gastrointest Liver Physiol.

[46] Wang X, Ren Q, Wu T, Guo Y, Liang Y, Liu S (2014). Ezetimibe prevents the development of non-alcoholic fatty liver disease induced by high-fat diet in C57BL/6J mice. Mol Med Rep.

[47] Shimada M, Shimano H, Gotoda T, Yamamoto K, Kawamura M, Inaba T, Yazaki Y, Yamada N (1993). Overexpression of human lipoprotein lipase in transgenic mice. Resistance to diet-induced hypertriglyceridemia and hypercholesterolemia. J Biol Chem.

[48] Kitajima S, Morimoto M, Liu E, Koike T, Higaki Y, Taura Y, Mamba K, Itamoto K, Watanabe T, Tsutsumi K, Yamada N, Fan J (2004). Overexpression of lipoprotein lipase improves insulin resistance induced by a high-fat diet in transgenic rabbits. Diabetologia.

[49] Poulsen LI, Siersbæk M, Mandrup S (2012). PPARs: fatty acid sensors controlling metabolism. Semin Cell Dev Biol.

[50] Amirullah NA, Zainal Abidin N, Abdullah N (2018). The potential applications of mushrooms against some facets of atherosclerosis: a review. Food Res Int.

[51] Zhong D, Xie Z, Huang B, Zhu S, Wang G, Zhou H, Lin S, Lin Z, Yang B (2018). *Ganoderma lucidum* polysaccharide peptide alleviates hepatoteatosis via modulating bile acid metabolism dependent on FXR-SHP/FGF. Cell Physiol Biochem.

[52] Wang X, Shi L, Joyce S, Wang Y, Feng Y (2017). MDG-1, a potential regulator of PPARα and PPARγ, ameliorates dyslipidemia in mice. Int J Mol Sci.

[53] Yang Z, Liu G, Wang Y, Yin J, Wang J, Xia B, Li T, Yang X, Hou P, Hu S, Song W, Guo S (2019). Fucoidan A2 from the brown seaweed *Ascophyllum nodosum* lowers lipid by improving reverse cholesterol transport in C57BL/6J mice fed a high-fat diet. J Agric Food Chem.

[54] Liu Q, Siloto RM, Lehner R, Stone SJ, Weselake RJ (2012). Acyl-CoA: diacylglycerol acyltransferase: molecular biology, biochemistry and biotechnology. Prog Lipid Res.

[55] Jin L, Shi X, Yang J, Zhao Y, Xue L, Xu L, Cai J (2021). Gut microbes in cardiovascular diseases and their potential therapeutic applications. Protein Cell.

[56] Romero-Córdoba SL, Salido-Guadarrama I, Meneses ME, Cosentino G, Iorio MV, Tagliabue E, Torres N, Sánchez-Tapia M, Bonilla M, Castillo I, Petlacalco B, Tovar AR, Martínez-Carrera D (2020). Mexican *Ganoderma Lucidum* extracts decrease lipogenesis modulating transcriptional metabolic networks and gut microbiota in C57BL/6 mice fed with a high-cholesterol diet. Nutrients.

