# Supplementary material for: Polysaccharide CM1 from Cordyceps militaris hinders adipocyte differentiation and alleviates hyperlipidemia in LDLR(+/−) hamsters
Source: Lipids Health Dis. 2021 Dec 13;20:178. doi: 10.1186/s12944-021-01606-6 (PMC8667404; doi:10.1186/s12944-021-01606-6)
Supplement: Supplementary file 1 — Additional file 1. [file 12944_2021_1606_MOESM1_ESM.docx]

Western blot for the Plasma part in Figure 2E-G.


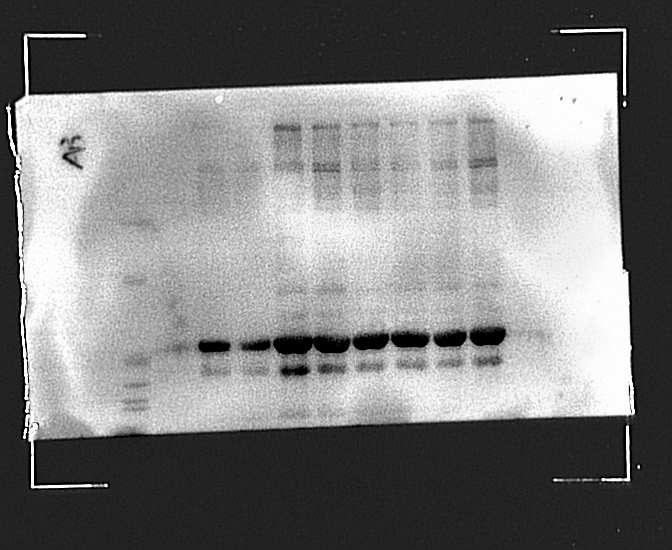


Albumin


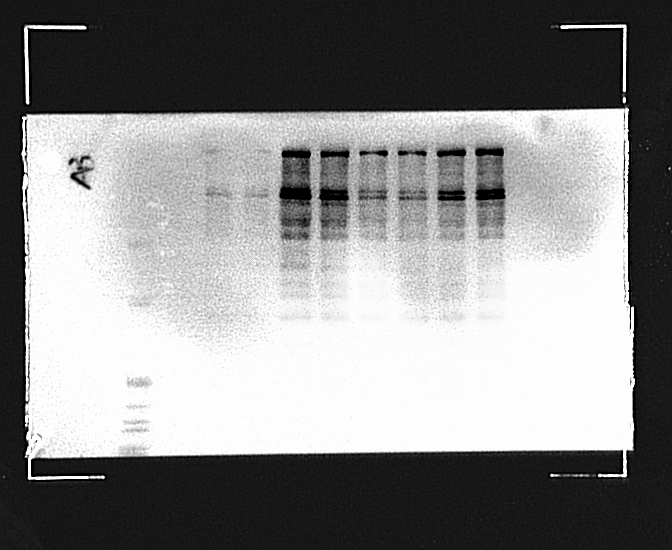


apoB


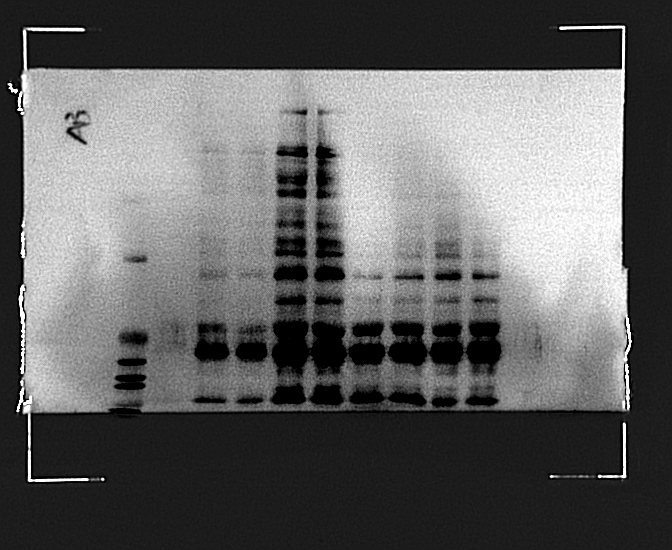


apoAI (The down bands)


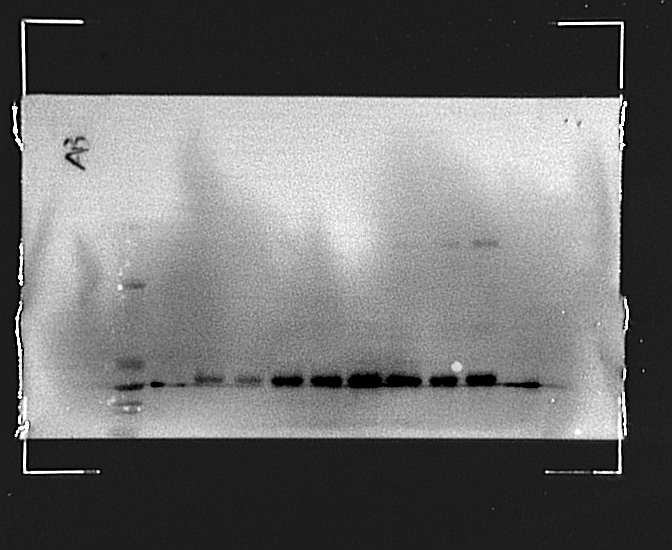


LPL

The bands of apoAI, apoB, LPL, and albumin were obtained from the same membrane (as labelled “A3” in the top-left corner) by blotting with different antibodies.
